# Placenta Prævia

**Published:** 1858-07

**Authors:** J. P. Terrell

**Affiliations:** Mackinaw, Ill.


ARTICLE II.

### PLACENTA PRÆVIA.

BY J. P. TERRELL, M. D., OF MACKINAW, ILL.

I was called, March 2d, 1858, in the country a few miles, to see a Mrs. W., who was reported to be in a dying condition, from loss of blood. On arriving at the house, I was immediately introduced into the sick chamber of my patient. Her face looked bloodless, pulse failing rapidly, regular labor pains, each pain accompanied with a gush of blood; said she had commenced wasting a month ago, but did not suppose she was in a dangerous condition; said she was about seven months gone.

I made an examination per vaginum, and learned that I had to do with a case of placenta prævia. The os uteri was dilated to about the size of a fifty cent piece, and rigid. The placenta was presenting directly over os uteri.

I informed the husband of the dangerous condition of his wife, and requested him to send for some other doctor; but there was none called, for reasons that I need not here state.

The life of my patient was running rapidly away, and something must be done, and done quickly, or else her life would be soon run out, and the scene would be closed.

I used all the means and appliances within my reach to hasten dilatation and check the hemorrhage; but the only thing I did that was of any benefit, was elevating the foot of the bed, which appeared to exert a slight influence over the hemorrhage.

About this time, my patient informed me that she had not felt any movements of the child for many hours. I immediately auscultated the abdominal tumor, and was soon satisfied that the child had ceased to live. But of what benefit would this discovery be to my patient? The uterus was still contracting a little, but her strength was rapidly on the decline. The child was dead, and so it claimed no part of my attention, at least for the present. I asked myself why, If the placenta was entirely detached, the head of the child would not come down and arrest the hemorrhage? I accordingly introduced my hand, and with my index and middle fingers, succeeded in entirely detaching the placenta from the uterus, and also discharging the bag of waters.

I kept the placenta pushed to one side as much as possible, so that when a pain did come on, the head might pass down and freely engage in the mouth of the womb, and check the hemorrhage. I was almost despairing of success, and was coming to the conclusion that I in all probability was hastening the fatal result.

But thank the fates, about this time the uterus commenced to contract strongly down upon its contents, the head was pressed down with considerable force, but I succeeded in keeping the placenta above and to one side of the os uteri, until the second pain came, and when, to my great joy, the head glided past and immediately became engaged in the os uteri, instantly checking the hemorrhage. I now for the first time had a spare minute to think. I found that I had been with my patient some two or three hours, and that the bright luminary of day was just going down beyond the western prairie, and ere long sable darkness would steal in and absorb the golden rays that were being shed over the eastern sky. But I dared not stand and watch the changing of the beautiful landscape before me, for if my patient was not bleeding to death, she was in a fainting, almost dying condition, and I must leave no means untried to save the life of the wife and mother entrusted to my care. No neglect of mine shall cause the husband and father to exclaim, "I am alone in the world, my best friend, the wife of my bosom is gone;" or the children to cry, "we are orphans, our mother is dead."

I gave her beef tea, opii and sugar lead, quinine and brandy, etc., through the night with, I thought, benefit, for in the morning (March 3d) she appeared to have more strength than on the previous evening. She remarked that she "felt quite strong this morning." She slept some through the day, had but little pain, until towards evening they began to grow stronger and and more frequent. The os was not so rigid, and I thought was dilating slowly. I gave her occasionally teas of different kinds, hoping they might be of some benefit. This condition of things continued, with gradually increasing pains, until eight o'clock, when I learned, to my great satisfaction, that all the parts were rapidly dilating and the labor progressing as safely as I could expect; and before twelve o'clock, with the aid of the forceps, my patient was delivered of quite a large child, which presented the appearance of having been dead for several days, and no less than a minute after the child was born I brought away the after-birth, put on the bandage, introduced my hand into the cavity of the womb, with my fingers irritated its walls, which caused it to contract, expelling my hand, and my patient was free from a hemorrhage that had again threatened her life.

*March 4th.* Wasting not too much, gaining strength slowly.

*March 5th.* Same as yesterday.

*March 6th.* Strength considerably improved, severe after-pains, has had no chill, used the catheter to draw off the urine, which gave some relief, gave her half an ounce castor oil.

*March 7th.* Found my patient in an alarming condition, had had a chill that morning, which lasted about an hour, after which a burning fever came on, pulse by the watch 120, feeble and intermittent, knees drawn up, abdomen slightly tympanitic, very tender on pressure, and severe abdominal pain. I directed a large mush poultice to cover the entire belly, and gave her the following:

Opii, grs. i.
Calomel, grs. vi.

One every four to six hours; tinct. veratrum viride, four drops every three hours. The powder was continued twenty-four hours, when free catharsis had occurred.

I then substituted the turpentine emulsion, of which I directed a teaspoonful, containing four drops of the veratrum, every three hours.

Under the above (being the principal) treatment she rapidly improved, and before six days had passed, I considered her clear of danger, so far as the fever was concerned.

Now many days since she is enjoying good health. There was no secretion of milk in this case.

I have had two cases of placenta prævia before, but in both I succeeded in turning and delivering without any great amount of trouble. But in the above case I could do no such thing. Some other course might have succeeded better, but I did just what occurred to my mind at the moment, without stopping to think whether or not I had ever seen anything written upon the subject.

